# Adaptive radiotherapy and the dosimetric impact of inter- and intrafractional motion on the planning target volume for prostate cancer patients

**DOI:** 10.1007/s00066-020-01596-x

**Published:** 2020-03-10

**Authors:** Felix Böckelmann, Florian Putz, Karoline Kallis, Sebastian Lettmaier, Rainer Fietkau, Christoph Bert

**Affiliations:** Department of Radiation Oncology, Universitätsklinikum Erlangen, Friedrich-Alexander-Universität Erlangen-Nürnberg, Universitätsstraße 27, 91054 Erlangen, Germany

**Keywords:** Endorectal balloon, Cone beam computed tomography, Drinking protocol, Margin calculation

## Abstract

**Purpose:**

To investigate the dosimetric influence of daily interfractional (inter) setup errors and intrafractional (intra) target motion on the planning target volume (PTV) and the possibility of an offline adaptive radiotherapy (ART) method to correct larger patient positioning uncertainties in image-guided radiotherapy for prostate cancer (PCa).

**Materials and methods:**

A CTV (clinical target volume)-to-PTV margin ranging from 15 mm in LR (left-right) and SI (superior-inferior) and 5–10 mm in AP (anterior-posterior) direction was applied to all patients. The dosimetric influence of this margin was retrospectively calculated by analysing systematic and random components of inter and intra errors of 31 consecutive intermediate- and high-risk localized PCa patients using daily cone beam computed tomography and kV/kV (kilo-Voltage) imaging. For each patient inter variation was assessed by observing the first 4 treatment days, which led to an offline ART-based treatment plan in case of larger variations.

**Results:**

Systematic inter uncertainties were larger (1.12 in LR, 2.28 in SI and 1.48 mm in AP) than intra systematic errors (0.44 in LR, 0.69 in SI and 0.80 mm in AP). Same findings for the random error in SI direction with 3.19 (inter) and 2.30 mm (intra), whereas in LR and AP results were alike with 1.89 (inter) and 1.91 mm (intra) and 2.10 (inter) and 2.27 mm (intra), respectively. The calculated margin revealed dimensions of 4–5 mm in LR, 8–9 mm in SI and 6–7 mm in AP direction. Treatment plans which had to be adapted showed smaller variations with 1.12 (LR) and 1.72 mm (SI) for Σ and 4.17 (LR) and 3.75 mm (SI) for σ compared to initial plans with 1.77 and 2.62 mm for Σ and 4.46 and 5.39 mm for σ in LR and SI, respectively.

**Conclusion:**

The currently clinically used margin of 15 mm in LR and SI and 5–10 mm in AP direction includes inter and intra uncertainties. The results show that offline ART is feasible which becomes a necessity with further reductions in PTV margins.

**Electronic supplementary material:**

The online version of this article (10.1007/s00066-020-01596-x) contains supplementary material, which is available to authorized users.

## Introduction

Adaptive radiotherapy (ART) is an accepted method to treat patients with prostate cancer (PCa) since image-guided radiotherapy (IGRT) cannot compensate completely for patient-specific treatment variations [1, 2]. Offline and online ART strategies, such as offline planning target volume (PTV) modification, offline dose compensation and online plan adaption, have been introduced to diminish systematic and random errors [2, 3]. One of the first offline ART strategies was introduced by Yan et al. [4] using daily CT and Martinez et al. [5] by applying online portal imaging for re-optimizing treatment plans within the first week of the treatment scheme. They dealt with systematic errors generated by interfractional setup uncertainties and intrafractional target motion.

The importance of intrafractional target motion for external beam radiation treatments (EBRT) increases as treatment margins are reduced due to IGRT [6, 7]. Therefore, at the University Hospital Erlangen, patients treated for PCa receive planning computed tomography (CT) and radiotherapy treatment based on a filled bladder and endorectal balloon (ERB) protocol in order to minimize target motion and spare dose to organs at risk (OAR) [8–17]. Prior to treatment, PCa patients are first localized with kV/kV-orthogonal image pairs (OIP) based on the position of fiducial markers (FM). Afterwards kilovoltage (kV) cone beam CT (CBCT) is performed to match anterior rectal wall and ERB to the planning CT. Using daily CBCT for patient positioning is a widely understood method for ART procedures [2, 3, 18–22].

The aim of the present analysis was to investigate the dosimetric impact of the clinically used CTV-to-PTV margin by retrospectively assessing daily interfractional setup and intrafractional prostate motion uncertainties. Furthermore, a clinical ART approach was established to evaluate setup errors within the first treatment sessions for each patient by retrospectively looking at the daily acquired CBCTs. If larger positioning uncertainties were observed, a new planning CT, an adapted set of contours and an adapted treatment plan followed. To our knowledge, this offline intervention based on an ERB and filled bladder protocol has not been published in the literature before.

## Materials and Methods

### Patients

The data presented are of 31 patients diagnosed with intermediate- or high-risk PCa who were treated at the University Hospital Erlangen between July 2015 and September 2016. Eleven of these patients received radiotherapy as primary, i.e., definitive, treatment while the remaining 20 patients underwent radical prostatectomy before radiotherapy. The patients had a mean age of 71 ± 9 years and TNM stage of cT1 (6.5%)–cT2 (29%) for primary radiotherapy and pT2 (29%)–pT3 (35.5%) as well as R0 (*n* = 12) or R1 (*n* = 8) for postoperative patients. The average Gleason score was 7.1 and varied from 5 to 10. The PSA level ranged from ≤4 to >20 ng/ml with ≤4 (*n* = 4), >4 to ≤10 (*n* = 16), >10 to ≤20 (*n* = 6) and >20 (*n* = 3) (Table 1).

**Table 1:** Patient data, including age at treatment time, patients with localized PCa, TNM stage [42], Gleason score [43], D’Amico risk stratification [44] and PSA level

Variable	*n* = 31
*Indication for radiotherapy, n (%)*	
Primary treatment (%)	11 (35.5%)
Postoperative treatment	20 (64.5)
*Age (years)*	
Mean (range)	71 (62–80)
*TNM stage (n)*	
cT1	2
cT2	9
pT2	9
pT3	11
R0	12
R1	8
*Gleason score (n)*	
5–7	26
8–10	5
*D’Amico risk group (n)*	
Intermediate risk	9
High risk	21
*PSA level (ng/ml)*	
≤4	4
>4 to ≤10	16
>10 to ≤20	6
>20	3

*TNM* classification of malignant tumors; *PCa* prostate cancer; *RT* radiotherapy; *PSA* prostate-specific antigen

Biochemical failure was defined according to the Phoenix criteria. For instance, a rise by 2 ng/ml or above the nadir PSA would indicate a biochemical failure. No patient experienced biochemical failure having a median biochemical follow-up of 18 months ranging from 3–33 months [23, 24].

### Pretreatment protocol

Three Gold Anchor™ fiducial markers 0.28 mm in diameter and 10–20 mm in length (Naslund Medical AB, Vassvagen, Sweden) were implanted transperineally under ultrasound (US) guidance into apex, and base region of the prostate 5 ± 2.5 days prior to CT simulation (Sensation Open CT scanner, Siemens Healthcare, Erlangen, Germany). All patients had a planning CT (1 mm slice thickness, 120 kV voltage, 400 mA X‑ray tube current) in supine position immobilized with knee, leg, and head pads. A contrast agent (10 ml Ultravist, Bayer Vital GmbH, Leverkusen, Germany) was injected into the bladder for better visibility. One hour prior to imaging and each radiation treatment, patients had (a) enemas (Microlax, Johnson & Johnson GmbH, Neuss, Germany) and were (b) asked to defecate. In addition, the clinical protocol also required the patients to (c) drink 1 liter of water to ensure a constant bladder filling, and (d) an ERB (Rüsch AG, Kernen, Germany) was inserted to the rectum and inflated with 65 cm^3^ of air.

### Contouring and treatment planning

OARs, i.e., rectum, anterior rectal wall, bladder, femoral heads, CTV and PTV were defined on the planning CT by radiation oncologists using iPlan Radiotherapy (RT) Image 4.1.1 (BrainLAB AG, Munich, Germany). For definitive radiotherapy, the PTV was contoured as described previously by Lettmaier et al. [25]. Briefly, the clinical target volume (CTV) consisted of the prostate and seminal vesicles as defined on the planning MRI (magnetic resonance imaging). For the PTV, 15 mm were added to the CTV in all directions. Posteriorly, the PTV was limited to one half of the anterior rectal wall to spare the rectal mucosa, which is equivalent to a 5–10 mm margin extension. For postoperative patients, CTV definition was adapted from the RTOG consensus guidelines [26]. In brief, inferiorly the CTV extended to the genitourinary diaphragm and superiorly included the vas deferens or seminal vesicle remnants, lateral boundaries were the levator ani and the internal obturator muscles, respectively. The anterior boundary was marked by the posterior edge of the pubic bone and the posterior bladder wall. Posteriorly, the CTV extended to the anterior rectal wall. As in definitive cases, the resulting PTV was limited posteriorly to one half of the anterior rectal wall to spare the rectal mucosa.

**Fig. 1:**
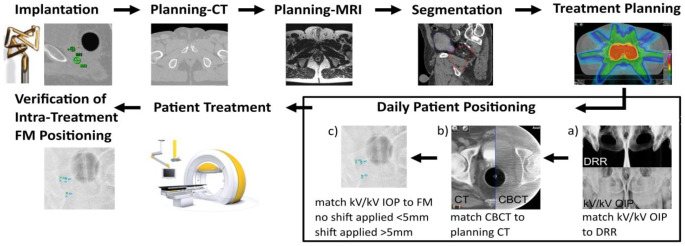
The scheme of clinical steps involving FM (fiducial markers) implantation, CT (computed tomography) and MRI (magnetic resonance imaging), segmentation, treatment planning, patient setup, patient treatment and verification of intratreatment FM positioning. Example of patient positioning in detail are shown in *a)*, *b)* and *c)*. *a)* Merged kV/kV OIP (orthogonal image pairs) to the DRR (digitally reconstructed radiograph) of the planning CT based on bony landmarks. *b)* Matching results of CT and CBCT (cone beam computed tomography) in axial plane. Special care was taken to ensure the correct location of anterior rectal wall and the ERB in both image sets. *c)* Registered kV/kV-OIP to planning CT based on FM location. Shift is applied when FM alignment exceeds 5 mm

All patients received a 7-field step-and-shoot intensity-modulated radiation therapy (IMRT) with a dose of 50.4 Gy (1.8 Gy/fraction) and were treated at the Vero® system (Mitsubishi Heavy Industries, Ltd., Tokyo, Japan and BrainLAB AG) using iPlan RT dose 4.5.3 as treatment planning system (TPS). The dose constraints are described in Supplementary I Table 1 and a standard 7‑field IMRT dose distribution in axial, coronal and sagittal plane can be seen in Supplementary I Fig. 1 (a, b, c).

### Patient setup

Before each treatment, patients were positioned using an image guidance system (ExacTrac®, BrainLAB AG, Munich, Germany) consisting of two kV tubes (Shimadzu Corp., Kyoto, Japan) and two amorphous silicon detectors (PaxScan 4030A; Varian Medical Systems, Palo Alto, CA, USA). The system was used to acquire a kV/kV-OIP and a volumetric CBCT data set using a clockwise rotation (315–45°) of one kV-imager with the following parameters: ~100 kV, 100 mA, 5 s; 3 mm slice thickness and 512 × 512 matrix size. The field of view (FOV) is restricted to 200 mm in diameter and 150 mm in length [27, 28]. All patients were first arranged to skin markers. Subsequently, the acquired kV/kV-OIP were merged to the DRRs (digitally reconstructed radiograph) of the planning CT according to bony landmarks (Fig. 1a). Possible translational and rotational misalignments were corrected by the treatment couch. Furthermore, the CBCT image was rigidly registered to the planning CT based on anterior rectal wall and ERB information by experienced radiation therapists (Fig. 1b). Before treatment, another kV/kV-OIP was taken to verify a FM mispositioning of below 5 mm, otherwise another table shift would have been performed to reduce the patient setup error (Fig. 1c). Prior to the last field of the treatment radiation, a kV/kV-OIP was performed to locate the FM for further intrafractional prostate motion analysis.

The full sequence of clinical steps starting with FM implantation and followed by CT and MRI imaging, segmentation, treatment planning, patient setup, patient treatment and lastly verification of intratreatment FM positioning is summarized in Fig. 1.

### Adaptive plan procedure

**Fig. 2:**
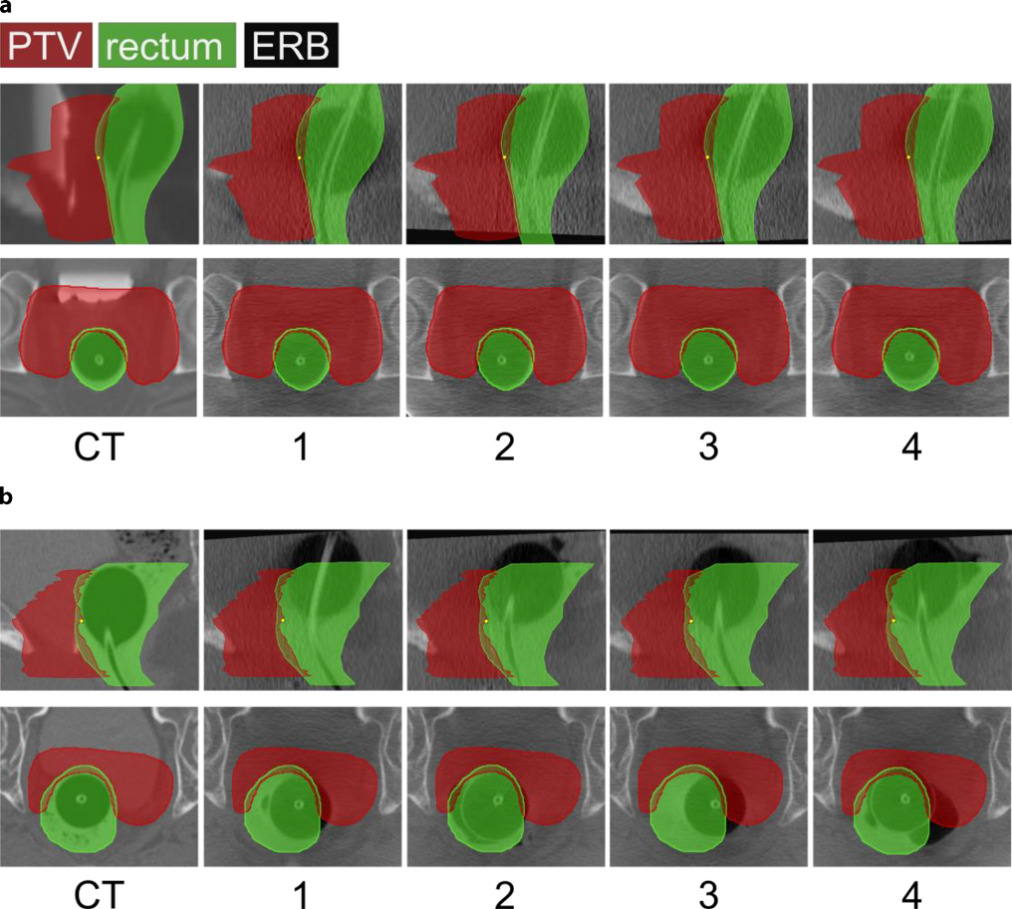
Example of patient positioning for the first 4 treatment days. The planning CT (computed tomography) on the far left represents the reference for the following CBCTs rigid registration. In **a**, a patient with a low setup error and in **b** a patient corresponding to larger positioning variation are displayed. For **b** offline ART was applied. *PTV* planning target volume, *ERB* endorectal balloon

The first four fractions were used to inspect the patient positioning by comparing planning CT and CBCTs by radiation oncologists and medical physicists. Fig. 2a displays a high CT/CBCT agreement, which means the ERB and anterior rectal wall exhibited small shifts but were within the defined rectal volume (green contour) structure. For this case PCa patients were not associated with an adapted treatment plan. If the setup errors revealed that the ERB and anterior rectal wall are outside of the rectal volume delineation, i.e., the patient subject to larger setup uncertainty as shown in Fig. 2b, a new planning CT, an adapted set of contours, and an adapted treatment plan followed. For critical cases, such as patients with larger setup uncertainty, setup and intrafraction kV/kV-OIPs were looked at additionally along with CBCTs to better understand how well FM, ERB and rectal wall were correlating with each other.

### Evaluation scheme

**Fig. 3:**
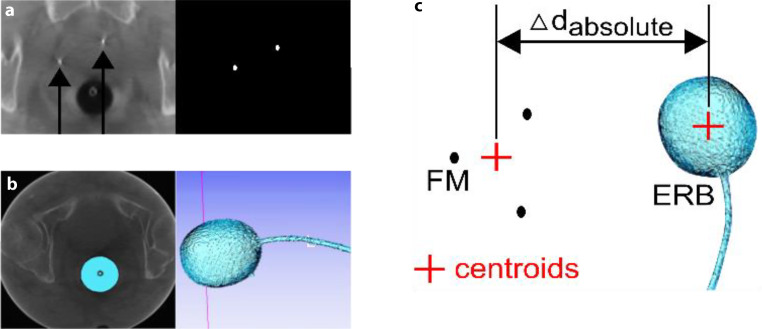
Example of data acquisition obtained in this study. In **a**, left and right CBCT and ITK-based images represent the marker extraction, whereas the images in **b** indicate a segmented ERB (*light blue*). On the *left image* in **a**, the *black errors* demonstrate the position of FM (fiducial markers) in the planning CT and on the *right* the location of FM are displayed as *white dots* in the binary image. Image **c** describes how to assess the absolute distance (d_absolute_) between the FM and ERB (endorectal balloon) centroids (*red crosses*) which defines the reference CBCT of a cohort of 28 CBCTs for each patient

An in-house software tool based on Insight Segmentation and Registration Toolkit (ITK) was used to quantify interfractional patient setup errors according to ERB displacements in CBCTs. This tool segmented the ERB and FM of each CBCT as well as the planning CT. It also assessed the geometrical arrangements of FM and investigated the shape, position and centroid location of ERB. Fig. 3a shows an example of a FM CBCT segmentation, whereas Fig. 3b indicates the same for the ERB. For more details about the ERB and FM segmentation, please refer to Supplementary II Segmentation of ERB and FM.

Interfractional left–right (LR), superior–interior (SI) and anterior–posterior (AP) shifts were analyzed by comparing a reference CBCT to the remaining 27 CBCTs acquired for each patient (1 CBCT/fraction equals 28 CBCTs in total). The reference CBCT was identified using the location of FM and ERB centroids which were defined in each CBCT and the planning CT. The centroid of the FMs was calculated by taking the mean of x, y and z coordinates and the centroid of ERB was extracted by using the in-house software tool. Then the absolute distance (∆d_absolute_) between the two centroids (FM and ERB) was examined in each data set (CT and CBCT), as illustrated in Fig. 3c. The absolute distance (∆d_absolute_) had to be below 1 mm between the planning CT and at least one CBCT data set to quantify high geometrical accuracy and to fulfill the condition for further analysis. The set of CBCT which had the lowest absolute distance of all 28 CBCTs and met the departmental requirements was selected as reference CBCT. Each patient’s reference CBCT and its absolute distance (∆d_absolute_) is represented in Supplementary I Fig. 2. The group systematic deviations (μ), standard deviation (SD), systematic deviation (Σ_inter_) and random deviation (σ_inter_) were calculated to investigate LR, SI and AP motion between the reference CBCT and the cohort of 27 CBCTs for each patient. More details about how the group systematic deviations (μ), standard deviation (SD), systematic deviation (Σ_inter_) and random deviation (σ_inter_) were derived can be found in section Supplementary III—Definition of statistics.

To determine intrafractional FM motion in LR, SI and AP direction, kV/kV-OIPs were taken after patient setup and prior to the last field of treatment for each fraction and each patient. For statistical analysis μ, SD, Σ_intra_ and σ _intra_ were used to assess LR, SI and AP motion.

Additionally, for a subcohort of 11 definitive PCa patients, the dosimetric impact of daily interfractional ERB setup errors and intrafractional FM motion on the PTV coverage and OAR dose was investigated by comparing treatment plans as per TPS (i.e., the clinically applied plan with no additional motion applied) against treatment plans based on daily inter- and intrafractional motion. The treatment plans with motion included were generated by shifting the isocenter according to daily inter- and intrafractional variation. Interfractional LR, SI and AP shifts were taken from CBCT data, as described above. Intrafractional motion data were used from acquired pre- and posttreatment kV/kV-OIPs, as discussed previously. The treatment plans as per TPS and inter- and intrafractional errors were assessed by using the in-house software tool to generate treatment plans based on daily inter- and intrafractional variations. The latter data were summarized in a dose–volume histogram (DVH).

Also, a CTV-to-PTV margin was calculated to estimate its magnitude when daily setup errors and intrafractional motion was applied and compared against the CTV-to-PTV margin which was used for treatment planning. First, the total Σ and σ were defined as Σ = (Σ^2^_inter_ + Σ^2^_intra_)^1/2^ and σ = (σ^2^_inter_ + σ^2^_intra_)^1/2^ [6]. Second, the formulas of Stroom et al. with margin = 2.0Σ + 0.7σ and van Herk et al. with margin = 2.5Σ + 0.7σ were used to estimate the CTV-to-PTV extension. More details about the margin calculation of Stroom et al. and van Herk et al. can be found in Supplementary IV—Margin recipe.

Regarding patients which followed the adaptive plan procedure, initial and adapted treatment plans were compared to determine the magnitude of interfractional ERB setup error by using μ, SD, Σ_inter_ and σ_inter_ for LR, SI and AP motion. A CTV-to-PTV margin was calculated for initial and adapted plans by applying first Oehler et al. [6] and subsequently Stroom et al. [29] as well as van Herk et al. [29] formulas to examine the impact of adapted treatments.

### Statistical analysis

In this study the similarity of prostate, anterior rectal wall, and rectum dose coverage influenced by inter- and intrafractional motion was statistically evaluated to the treatment plan as per TPS. A nonparametric Wilcoxon test with paired samples was used with a significance level of *p* = 0.05. The reason for this choice was to compare the variance of the group mean error (µ) of two samples. This test requires the data to be normally distributed. Most smaller samples fulfill this criterion. Larger samples even if they are nearly normal distributed fulfill this criterion as well [30]. Prior to statistical evaluation, the data were examined for extreme outliers which would cause the sample to not be normally distributed. Using IGRT for daily patient setup, extreme outliers were not to be expected in the dataset. The sample size was 119 which is equivalent to a resolution of one data point per 0.5 Gy and may correspond to a larger sample size [31].

## Results

Table 2 represents interfractional setup errors of ERB and intrafractional prostate motion uncertainty in LR, SI and AP direction as well as PTV margin calculation involving a cohort of 31 patients (868 CBCTs, 1736 kV/kV-OIPs and 31 planning CTs). The shifts in SI direction for interfractional setup uncertainties showed the largest magnitude for Σ with 2.28 mm (LR with 1.12 mm and AP with 1.48 mm) and σ with 3.19 mm (LR with 1.89 mm and AP with 2.1 mm). The systematic error was lower for intrafractional motion than for interfractional patient positioning (LR with 0.44 mm and 1.12 mm, SI with 0.69 mm and 2.28 mm and AP with 0.80 mm and 1.48 mm), whereas random errors were alike in LR and AP direction (LR with 1.91 mm and 1.89 mm and AP with 2.27 mm and 2.10 mm). The mean group deviation (μ) varies near zero for all inter- and intrafractional data with standard deviations ranging from 2 to 4 mm. The CTV-to-PTV margin estimation reveals dimensions of 4–5 mm in LR, 8–9 mm in SI and 6–7 mm in AP direction.

**Table 2:** The results of interfractional setup errors based on ERB CT/CBCT co-registration and the outcome for intrafractional motion errors indicated by FM matching using CT and kV/kV-OIP images for 31 patients. Additionally, a CTV-to-PTV margin calculation is represented by using the data of 31 patients

		Translation in mm	LR	SI	AP
CT/CBCT:	ERB	Interfraction setup error	–	–	–
		μ	−0.22	0.42	−0.03
		SD	2.03	4.00	2.53
		Σ inter	1.12	2.28	1.48
		σ inter	1.89	3.19	2.1
CT/kV/kV-OIP:	FM	Intrafraction motion error	–	–	–
		μ	0.04	−0.28	−0.70
		SD	1.95	2.43	2.36
		Σ intra	0.44	0.69	0.80
		σ intra	1.91	2.30	2.27
CTV-PTV margin calculation:	ERB + FM	–	–	–	–
Oehler et al. [6]		Σ	1.20	2.38	1.68
		σ	2.23	3.93	3.09
Stroom et al. [36]		–	3.97	7.52	5.52
Van Herk et al. [29]		–	4.57	8.71	6.37

*LR* left–right; *SI* superior–inferior; *AP* anterior-–posterior; *μ* group systematic deviations; *SD* 1 standard deviation; *Σ* systematic deviation (1 standard deviation); *σ* random deviation (1 standard deviation); *kV/kV-OIP* kilovoltage/kilovoltage orthogonal image pairs; *CT* computed tomography; *CBCT* cone beam computed tomography

**Fig. 4:**
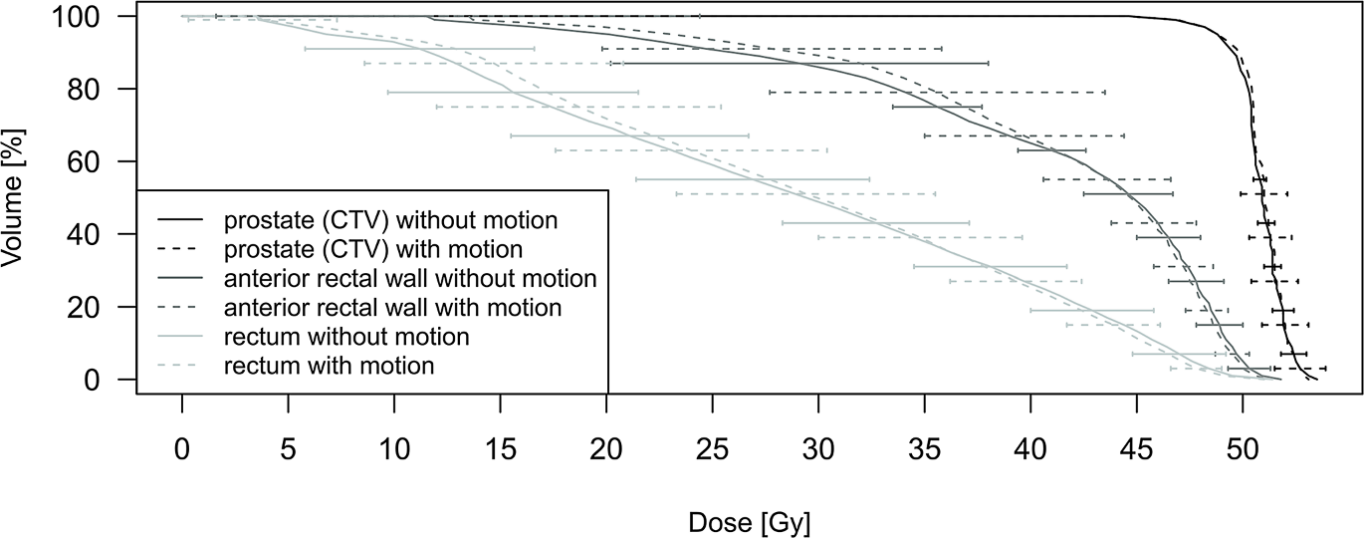
The dose–volume histogram displays the mean dose (Gy) for prostate (CTV), anterior rectal wall, and rectum volumes (%) affected by inter- and intrafractional motion (*dotted line*) and the original treatment plan (*solid line*) for 11 patients with primarily definitive radiotherapy. The error bars indicate the variation (1 standard deviation) of the dose for each volume. *GTV* gross target volume, *FM* fiducial markers, *ERB* endorectal balloon

The DVH in Fig. 4 shows the mean dose of prostate (CTV), anterior rectal wall, and rectum volumes. Each volume is represented by two DVH curves. One is influenced by (inter- and intrafractional) motion and the other one is based on the initial plan. Both DVHs are compared against each other and involve data of 11 patients with definitive radiotherapy (308 CBCTs, 616 kV/kV-OIPs and 11 planning CTs). Differences were not statistically significant. The variation (1 standard deviation) of the DVH curves for prostate and rectum volumes demonstrated analogous results.

Six patients out of 31 patients (168 CBCTs, 6 initial planning CTs and 6 re-planned CTs) with larger setup uncertainties received offline ART. Interfractional patient positioning accuracy for initial and adapted treatment plans were examined as well as CTV-to-PTV margins were calculated which can be seen in Table 3. The shifts in LR and SI directions are smaller for the adapted plan (1.12 and 1.72 mm for Σ and 4.17 and 3.75 mm for σ) than for the initial plan (1.77 and 2.62 mm for Σ and 4.46 and 5.39 mm for σ). In terms of AP motion, adapted (1.73 mm for Σ and 3.20 for σ) and initial (1.67 mm for Σ and 3.21 for σ) plans were observed to be alike. For μ values, no clear correlation between initial and adapted plans was determined (1.36 ± 4.56, 0.24 ± 6.4 and −0.28 ± 3.61 mm and −0.32 ± 3.06, −1.46 ± 4.87 and 0.95 ± 3.59 mm). The CTV-to-PTV margin in AP direction showed similar outcome ranging 6–8 mm comparing initial and adapted plans, whereas LR and SI margins of adapted plans appear to be 2 mm (5–6 mm and 7–8 mm) and 3 mm (7–8 mm and 10–11 mm) smaller than those based on the initial plans.

**Table 3:** Summary of setup errors and CTV-to-PTV margin computation for 6 patients where offline ART was performed on

			Initial plan			Adapted plan		
Translation in mm			LR	SI	AP	LR	SI	AP
Interfraction setup error	ERB	–	–	–	–	–	–	–
		μ	1.36	0.24	−0.28	−0.32	−1.46	0.95
		SD	4.56	6.4	3.61	3.06	4.87	3.59
		Σ inter	1.77	2.62	1.67	1.12	1.72	1.73
		σ inter	4.46	5.39	3.21	4.17	3.75	3.20
CTV-PTV margin calculation:	ERB + FM	–	–	–	–	–	–	–
Oehler et al. [6]		Σ	1.82	2.71	1.85	1.20	1.85	1.91
		σ	4.62	5.86	3.93	4.34	4.40	3.92
Stroom et al. [36]		–	6.88	9.52	6.46	5.44	6.79	6.56
Van Herk et al. [29]		–	7.79	10.88	7.38	6.04	7.71	7.51

*LR* left–right; *SI* superior–inferior; *AP* anterior–posterior; *μ* group systematic deviations; *SD* 1 standard deviation; *Σ* systematic deviation (1 standard deviation); *σ* random deviation (1 standard deviation)

## Discussion

IGRT addresses patient positioning errors but cannot completely compensate for patient-specific variations [32]. ART is the ideal intervention to account for specific interfractional discrepancy, as it has been reported that ART correction strategies reduce systematic and random errors [1, 2, 22, 32]. In this article, systematic and random components of interfractional setup and intrafractional motion errors of 31 localized PCa patients were investigated. The dosimetric influence of inter- and intrafractional motion on the PTV was analyzed for 11 patients with primarily definitive radiotherapy. Furthermore, in 6 out of 31 patients an offline ART method was used clinically and data were evaluated with regards to interfractional variations.

The ERB contributes to rectal wall dose sparing by keeping the posterior wall distant from the high dose region, which is one reason for choosing ERB for our clinical workflow and it is widely applied in the literature [6, 15, 33]. In the present research, the interfractional ERB discrepancy showed the largest magnitude for systematic and random errors in SI direction (2.28 mm and 3.19 mm, respectively), which is an indication of daily placements depth variation [16, 34].

Smeenk et al. [35] examined that sporadically stool trapped by the ERB and muscular contraction and relaxation of patients after their positioning increases the interfraction setup error of the prostate location, but it is not related to intrafractional motion. In the present article, a larger systematic error for interfractional setup error and a lower systematic error for intrafractional motion was obtained and confirmed Smeenk et al. [35] findings (Table 2).

A clinically used CTV-to-PTV margin of 15 mm in LR and SI and 5–10 mm in AP direction is applied [25]. However, calculating a PTV margin involving interfractional setup and intrafractional motion errors according to the mathematical models of van Herk et al. [29] and Stroom et al. [36], 4–5 mm, 8–9 mm and 6–7 mm in LR, SI and AP direction, respectively, can be assumed. Indeed, the size of the initial CTV-to-PTV margin is larger than the calculated one. This can be observed in Fig. 4 where the dosimetric influence on prostate (CTV) and OAR volumes was investigated. The treatment plan as per TPS did not significantly differ to the one involving inter- and intrafractional variation, which indicates that inter- and intrafractional errors did not have an impact on the dose distribution of prostate and OAR volumes.

There are several approaches to pursue offline ART. McVicar et al. [2] summarized offline correction as PTV modification and dose compensation. It implies that systematic and random errors can be reduced by adopting patient setup or by calculating a cumulative dose for each treatment fraction using the “anatomy of the day”. The present article investigated offline ART accounting for large interfractional setup errors based on ERB and anterior rectal wall registration using CBCT for the first 4 treatment days. Offline ART shows a drop of systematic and random errors in LR and SI directions by adapting the contours and treatment plan according to a new planning CT, which met the expectation of other studies. Generally, reasons for setup complications were mild ERB discomfort and spontaneous stool and gas appearance. Spontaneous stool and gas appeared to be more severe for the 6 re-planned patients than for all the others and made daily precise positioning difficult. Still, most of the patients tolerated the insertion of ERB, which is also supported by several reports in the literature [8, 37].

In terms of calculating a CTV-to-PTV margin for initial and offline ART treatment plans, 7–8 mm, 10–11 mm and 6–8 mm and 5–6 mm, 7–8 mm and 6–8 mm in LR, SI and AP directions, respectively, could be expected. In the AP direction, the systematic and random errors were alike. This can be explained by matching the planning CT and CBCT during patient positioning. Throughout this procedure, special care was taken to align the anterior rectal wall contour in both images to reduce radiation-induced rectum toxicity. Additionally, the calculated CTV-to-PTV margin for initial and adapted plans would not have an impact on CTV (prostate) dose coverage since the initial CTV-to-PTV margin also compensates for larger daily uncertainties.

It can be argued for the reason of using an offline intervention looking at the data of this analysis, since the size of the initial PTV structure is large enough to cover positioning and treatment uncertainties. The aim in the future will be to gradually reduce the size of the PTV margin to be able to spare more dose to OAR and still keep the prescribed dose to the target volume [1, 4, 34, 38–40]. Minimizing the size of the PTV also means that inter- and intrafractional errors have a greater effect during treatment. Therefore, the feasibility of offline ART was introduced to prevent larger patient positioning errors having a larger impact on the future smaller PTV.

The present article neglected the rotational influence of prostate motion. There are several studies conducting intrafractional systematic and random motion for prostate rotation, which are summarized by McPartlin et al. [32]. They suggested that the importance of prostate rotation increases for small PTV margins and margins of 3 mm may be required to compensate for rotations up to 5°. Organ and ERB deformation were not considered in the analysis. ERB deformation may occur due to the existence of stool and gas alongside the ERB, inconsistent bladder filling and the use of neo-adjuvant hormone therapy [32, 34]. It has been reported that the deformation of the prostate can be up to 3 mm or 10–15% of the prostate volume [32]. Therefore, PCa patients, in this manuscript, were treated with a filled bladder and empty rectum to reduce the presence of stool and gas and to keep the size of the bladder uniform. Patients were not held on a strict diet during the treatment process, which may lead to the effect of spontaneous stool and gas complications and increased rectum and prostate shifts. Njikamp et al. [3] presented that the consequence of a strict diet reduces rectal volume variation and limit prostate motion. Furthermore, using a 100 cm^3^ air-filled ERB can have the same effect of decreasing rectum and prostate movements but might increase mild ERB discomfort to patients due to the larger size of the ERB [41]. The motion and dosimetric influence of the seminal vesicles was not analyzed but it may be the subject of a future article.

## Conclusion

Our current CTV-to-PTV margin of 15 mm in LR and SI and 5–10 mm in AP direction takes into account the inter- and intrafraction uncertainties. However, the PTV will be decreased in size and offline ART will play an important role in the future. In this manuscript, reducing systematic and random interfractional errors by applying offline ART based on adapting the contours and treatment plan according to a new planning CT was feasible.

## Caption Electronic Supplementary Material

Supplementary I. Table 1: Constraints used for 7-field IMRT optimization. The dose [%] is relative to the prescribed dose of 50.4 Gy. Supplementary I. Figure 1: Example of a standard 7-field IMRT dose distribution. The sagittal a), axial b) and coronal c) drawings demonstrate all three planes. Supplementary I. Figure 2: Summary of absolute distances (∆dabsolute) in [%] and [mm] of each patient’s reference CBCT. Every ∆dabsolute of the reference CBCT was subtracted by ∆dabsolute of the CT to determine the difference of both. The CT’s ∆dabsolute were considered as the baseline.

Supplementary II Segmentation of ERB and FM. Analysis of Marker Migration: In order to analyse possible motion of implanted gold markers, the inter marker distances (IMD) between three markers were evaluated. Estimation of Endorectal Balloon Variations: The reliability of the endorectal balloon (ERB) was evaluated using a segmentation pipeline based on ITK libraries and yielded information about the diameter, the size, the shape and the daily variations of the centre of gravity (position) of the ERB.

Supplementary III – Definition of statistics: In preparation for the data to be used for the margin recipes the systematic (Σ) and random error (σ) had to be calculated.

Supplementary IV – Margin recipe: Short summary about the history and the definition of the formulas of van Herk et al. and Stroom et al.
