# Supplementary material for: Adaptive radiotherapy and the dosimetric impact of inter- and intrafractional motion on the planning target volume for prostate cancer patients
Source: Strahlenther Onkol. 2020 Mar 10;196(7):647–56. doi: 10.1007/s00066-020-01596-x (PMC7305089; doi:10.1007/s00066-020-01596-x)
Supplement: Supplementary file 1 — Supplementary I. Table 1: Constraints used for 7-field IMRT optimization. The dose [%] is relative to the prescribed dose of 50.4 Gy. Supplementary I. Figure 1: Example of a standard 7-field IMRT dose distribution. The sagittal a), axial b) and coronal c) drawings demonstrate all three planes. Supplementary I. Figure 2: Summary of absolute distances (∆dabsolute) in [%] and [mm] of each patient’s reference CBCT. Every ∆dabsolute of the reference CBCT was subtracted by ∆dabsolute of the CT to determine the difference of both. The CT’s ∆dabsolute were considered as the baseline. [file 66_2020_1596_MOESM1_ESM.docx]

Supplementary I Table 1

Constraints used for 7-field IMRT optimization. The dose [%] is relative to the prescribed dose of 50.4 Gy.

|  | **Structure** | **Volumes** | **Dose criteria** | **Dose [Gy]/ [%]** |
| --- | --- | --- | --- | --- |
| 7-field IMRT: |  |  |  |  |
| Organ at risk: | Rectum | 2cc | Dmax | 52.0/ 103 |
|  | Anterior rectal wall | 0% | Dmax | 55.4/ 110 |
|  | Bladder | 0% | Dmax | 55.4/ 110 |
|  | Femoral heads | 0% | Dmax | 40.3/ 80 |
| Target volumes: | PTV | 95% | Dcoverage | 47.8/ 95 |
|  |  |  | Dmean | 50.4/ 100 |
|  |  | 0% | Dmax | 55.4/ 110 |

*Abbreviations:* IMRT= intensity-modulated radiation therapy; PTV= planning target volume; Dmax= Dose maximum; Dmean= mean Dose; Dcoverage= coverage dose; cc= [cm³].

Supplementary I Figure 1

Example of a standard 7-field IMRT dose distribution. The sagittal a), axial b) and coronal c) drawings demonstrate all three planes.


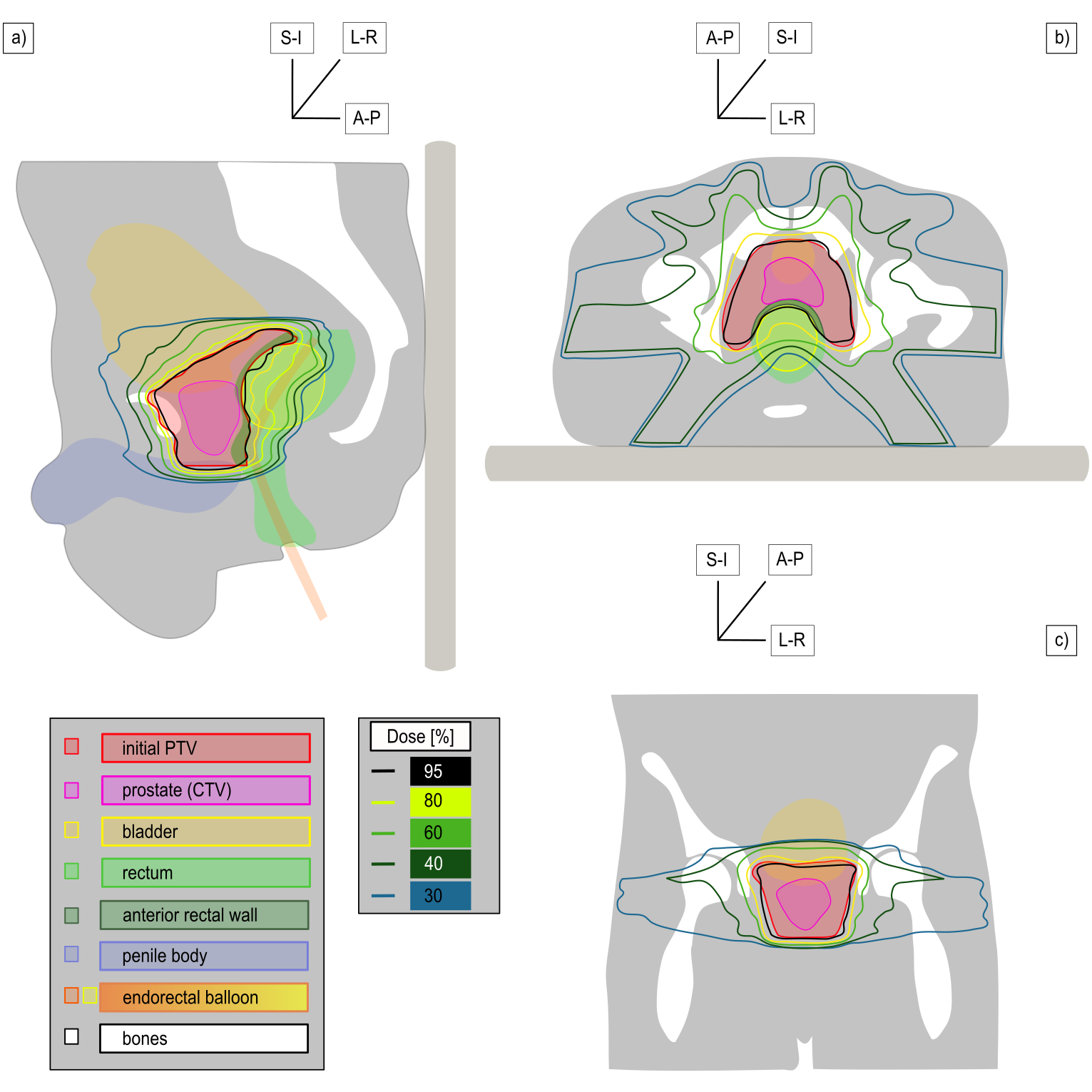


*Abbreviations:* LR= left-right; SI= superior-inferior; AP= anterior- posterior.

Supplementary I Figure 2

Summary of absolute distances (∆d_absolute_) in [%] and [mm] of each patient’s reference CBCT. Every ∆d_absolute_ of the reference CBCT was subtracted by ∆d_absolute_ of the CT to determine the difference of both. The CT’s ∆d_absolute_ were considered as the baseline.


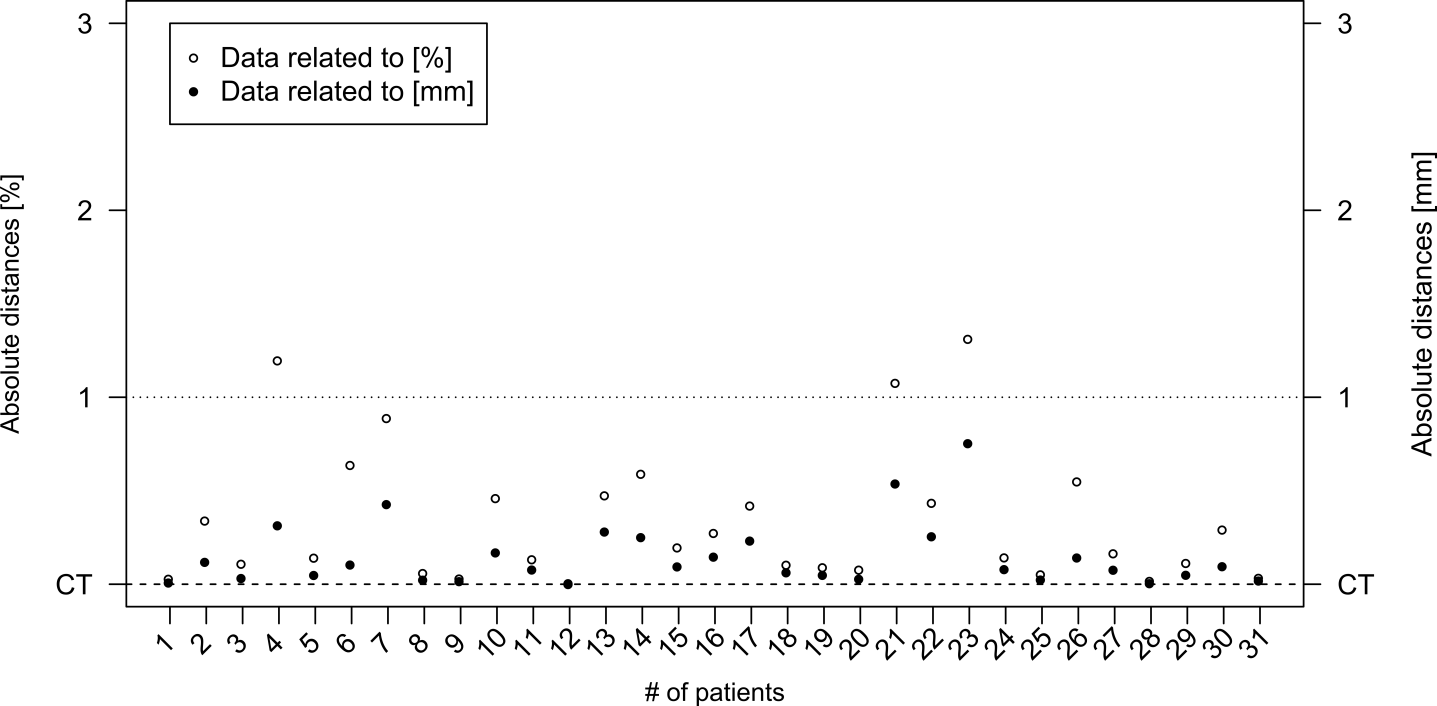


*Abbreviations*: CT=computed tomography; CBCT= cone beam computed tomography; #= number of patients.
