# Supplementary material for: Adaptive radiotherapy and the dosimetric impact of inter- and intrafractional motion on the planning target volume for prostate cancer patients
Source: Strahlenther Onkol. 2020 Mar 10;196(7):647–56. doi: 10.1007/s00066-020-01596-x (PMC7305089; doi:10.1007/s00066-020-01596-x)

Supplementary II Segmentation of ERB and FM

Analysis of Marker Migration

In order to analyse possible motion of implanted gold markers, the inter marker distances (IMD) between three markers were evaluated. The gold markers were placed around the prostate in a fixed manner, such that these three markers form a particular geometric arrangement shown in Supplementary II figure 1. In case of marker migration at least one marker moves to a different position and the distances between the surrounding markers changes. In order to evaluate the IMD out of various CBCT images a segmentation algorithm was introduced. The positions of the fiducial gold markers were detected automatically. The idea was to utilize the high-density material, the shape and the specific arrangement of the three of the fiducial gold markers to segment the region of interest. For this purpose, the Insight Segmentation and Registration Toolkit (ITK) was used.

Supplementary II Figure 2 shows the different segmentation steps. First, truncation artefacts were removed by cropping of the edges of the three-dimensional CBCT input image. The outcome is represented in Supplementary II Figure 2 b). Second, a threshold filter was applied. As a result of thresholding, a binary image (fiducial markers+ other high-density regions+ background) was produced. The HU values of metal depended on various image acquisition parameters and surroundings. Hence, the HU values varied between different patients and often between different fractions and reached intensity values up to 10000 HU. As a result, the threshold had to be flexible and was defined as the 99th percentile of the overall intensity value distribution of the cropped image. Third, each segmented region was extracted and removed if the geometric shape and size did not match the markers. Common errors for a wrong detection were caused by surgery clips or implanted seeds. The centre of gravity of each segmented fiducial marker was considered as the fiducial marker centroid.

Estimation of Endorectal Balloon Variations

The reliability of the endorectal balloon (ERB) was evaluated using a segmentation pipeline based on ITK libraries and yielded information about the diameter, the size, the shape and the daily variations of the centre of gravity (position) of the ERB. The CBCT image served as input for the segmentation pipeline. The first step was to crop off truncation artefacts as shown in Supplementary II Figure 3 b). The cropping size was chosen according to empirical values and was the same for all images. Since the balloon was filled with air, the intensity values of the region of interest were assumed to be between -1100 HU and -600 HU. Every intensity value outside of this interval was set to zero and the values within the range were set to 255. The outcome was a sparse representation of the ERB, shown in Supplementary II Figure 3 c). This resulted in a sparse segmentation of the ERB, which was used to randomly define four different seed points to run a more precise segmentation region growing algorithm. An assumption was made that the ERB would consist of at least 5000 voxels (which is equivalent to the maximum balloon capacity of 300 ml), every segmented region which would not fulfil this condition was removed. In case of multiple segmented regions separated from each other, the largest connected segment was assumed to be the ERB. As a result, a segmented smoothed ERB was acquired, observed in Supplementary II Figure 3 d). In order to calculating the centre of gravity of the ERB (ERB centroid), the volume of the object had to be computed which was defined as the number of voxels within the ERB times volume of one voxel.

Supplementary II Figure 1

Inter marker distance and geometric arrangement of gold markers is presented. If marker 1 moves away there is recognizable change in distance 1 and 3. The shifted marker 1 is called marker 1’.


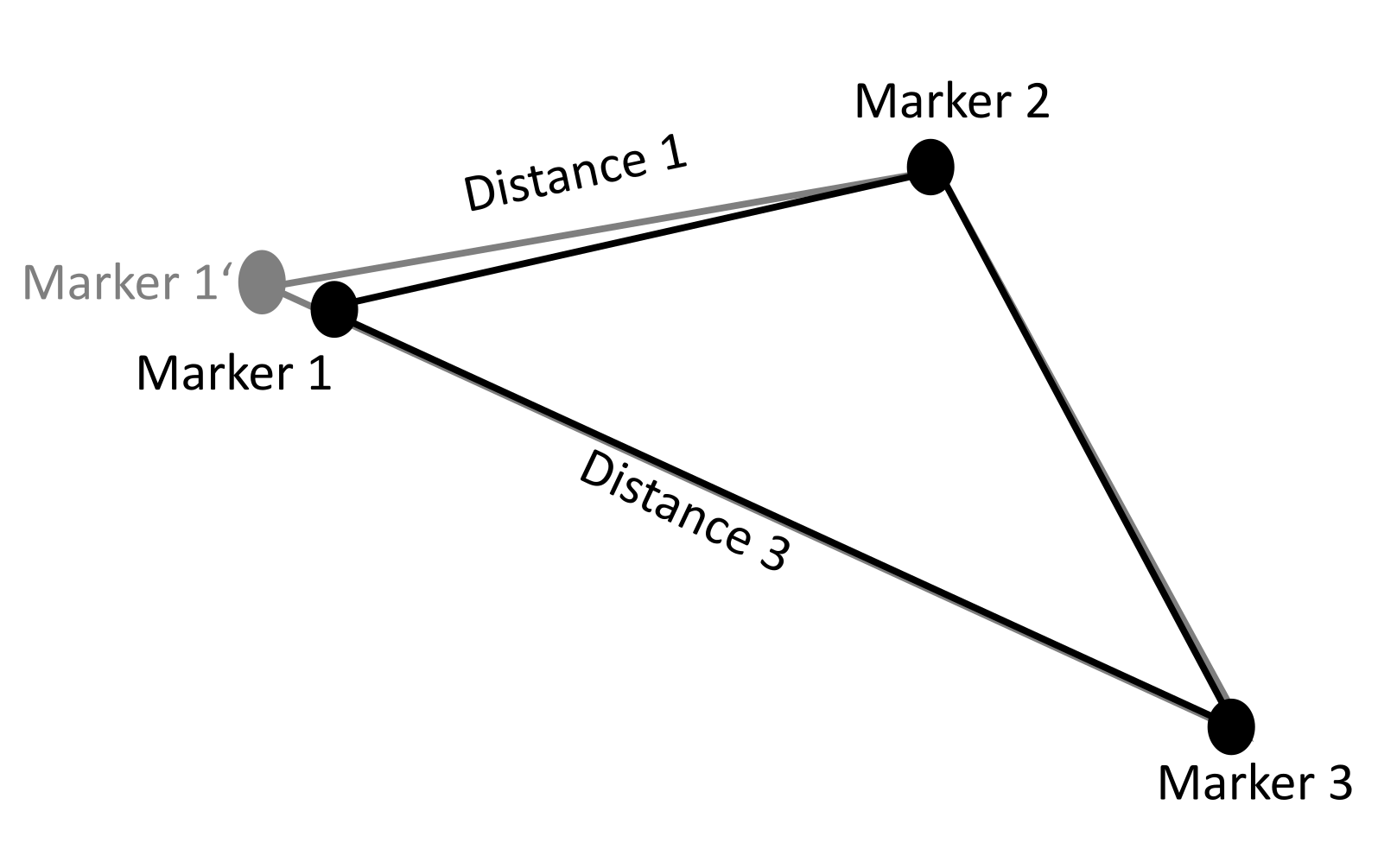


Supplementary II Figure 2

The segmentation pipeline of fiducial markers is represented in a), b) and c). a) Displays the initial CBCT, b) shows the cropped version of the CBCT and c) the binary image represents the segmented regions of the fiducial markers only.


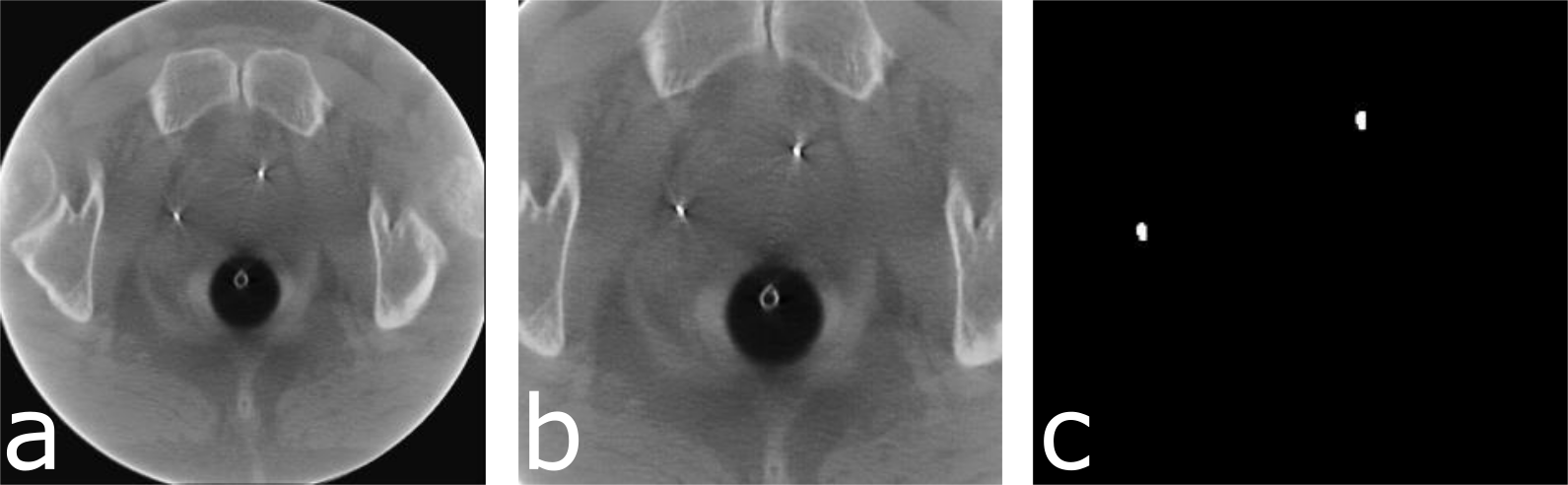


Supplementary II Figure 3

The segmentation pipeline of ERB is represented in a), b), c) and d). a) Displays the initial CBCT, b) the cropped version of the CBCT, c) shows the sparse segmented ERB including seed points to run the segmentation region growing algorithm and c) represents the extracted smoothed ERB.


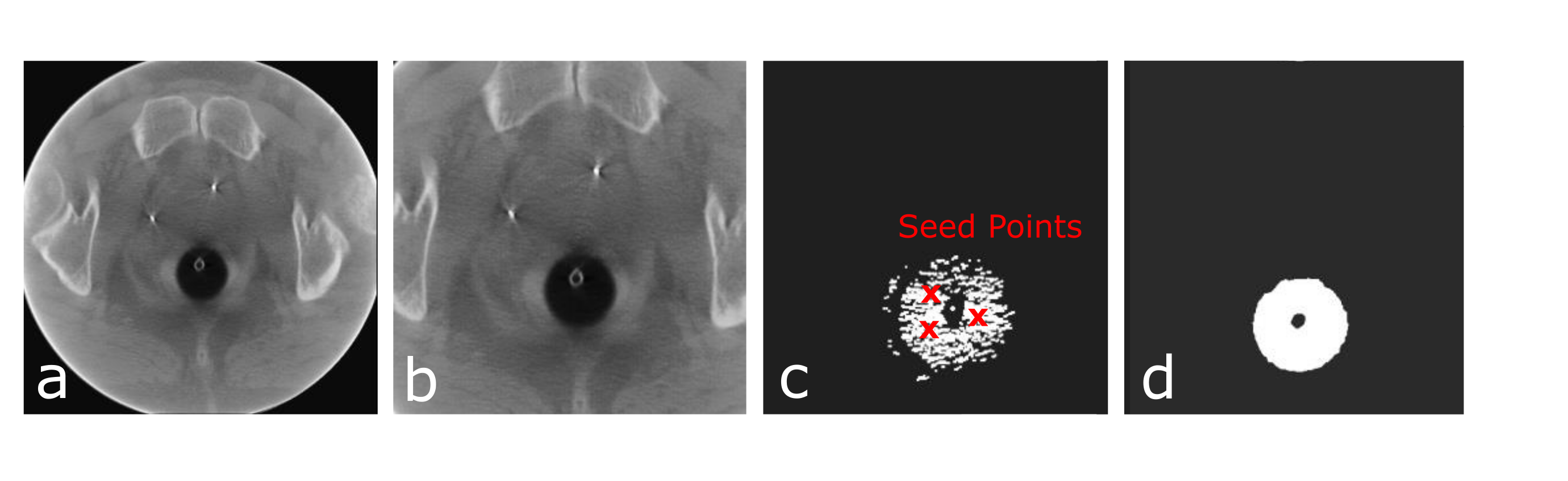

Supplement: Supplementary file 2 — Supplementary II Segmentation of ERB and FM. Analysis of Marker Migration: In order to analyse possible motion of implanted gold markers, the inter marker distances (IMD) between three markers were evaluated. Estimation of Endorectal Balloon Variations: The reliability of the endorectal balloon (ERB) was evaluated using a segmentation pipeline based on ITK libraries and yielded information about the diameter, the size, the shape and the daily variations of the centre of gravity (position) of the ERB. [file 66_2020_1596_MOESM2_ESM.docx]
