# Supplementary material for: Adaptive radiotherapy and the dosimetric impact of inter- and intrafractional motion on the planning target volume for prostate cancer patients
Source: Strahlenther Onkol. 2020 Mar 10;196(7):647–56. doi: 10.1007/s00066-020-01596-x (PMC7305089; doi:10.1007/s00066-020-01596-x)
Supplement: Supplementary file 3 — Supplementary III – Definition of statistics: In preparation for the data to be used for the margin recipes the systematic (Σ) and random error (σ) had to be calculated. [file 66_2020_1596_MOESM3_ESM.docx]

Supplementary III – Definition of statistics

In preparation for the data to be used for the margin recipes the systematic (Σ) and random error (σ) had to be calculated. The data consisting of 3 translational motion (LR, SI and AP) had to be split into the three components. For each component the group mean error ($m =\left( \sum x_{i} \right)/ n$) was determined over all fractions and patients and, subsequently, subtracted from all values. The result of that is the variance ($var=\left( m-i \right)$). Using the variance, the mean was computed for each patient over all fractions which resulted in the mean of the variance ($m_{var} =\left( \sum{var}_{i} \right)/ n$). Following to that the standard deviation was taken over all patients to derive the systematic error (∑=√((∑(x-$m_{var}$)^2)/(n-1))). In order to derive the random error first the variance was subtracted from the mean of the variance for each patient and second the standard deviation was taken over all patients (σ =√((∑(x-$\left( \sum(var-m_{var} \right)$)^2)/(n-1))).
