# Supplementary material for: Adaptive radiotherapy and the dosimetric impact of inter- and intrafractional motion on the planning target volume for prostate cancer patients
Source: Strahlenther Onkol. 2020 Mar 10;196(7):647–56. doi: 10.1007/s00066-020-01596-x (PMC7305089; doi:10.1007/s00066-020-01596-x)
Supplement: Supplementary file 4 — Supplementary IV – Margin recipe: Short summary about the history and the definition of the formulas of van Herk et al. and Stroom et al. [file 66_2020_1596_MOESM4_ESM.docx]

Supplementary IV – Margin recipe

A widely known margin recipe is the van Herk formula. This recipe is based on back in the days where IGRT wasn’t gold standard. Any deviation between the CT image and the daily patient positioning on treatment had an impact on the success of the treatment. Thus, a few requirements had to be made for the margin recipe to be considered as a guideline for radiotherapy. One aim was that the CTV-to-PTV extension should be big enough to cover up for deviations between the CT image and the patient positioning on treatment. Additionally, random (σ) and systematic (Σ) errors behave differently for fractionated treatments and that should be considered when calculating a safety margin. Systematic errors cause a shift of the dose distribution, whereas random errors induce a dose smearing. Additionally, systematic errors appear constant over the course of treatment and vary only from patient to patient. Examples of systematic errors can be contouring errors, patient positioning uncertainties or variance in organ motion during treatment preparation. Random errors vary in between fractions and during a patient’s treatment. They may arise from variations of internal organ, tumor or patient motion. Thus, the safety margin can be calculated as follows $M_{PTV}= \alpha\sum+\beta\sqrt{\sigma_{m}^{2}+\sigma_{p}^{2}}-\beta\sigma_{p}$, whereas $\sigma_{p}$ describes the penumbra width of the primary beam. α and β are factors to define the dose coverage. Using van Herk’s principle, the minimum dose to the CTV should be at least ≥ 95% of the prescribed dose for 90% of the patients, whereas for Stroom et al. 99% of the CTV volume should receive ≥ 95% of the prescribed dose. If the penumbra width is assumed to be $\sigma_{p}$= 3.2mm, the formula $M_{PTV}= \alpha\sum+\beta\sqrt{\sigma_{m}^{2}+\sigma_{p}^{2}}-\beta\sigma_{p}$ can be simplified to $M_{PTV}=2.5\sum+0.7\sigma$ according to van Herk et al.. It can be noticed that the systematic errors play a bigger role than random errors. That is because random errors usually compensate each other out over the course of treatment, whereas systematic errors increase the uncertainty of the treatment. The van Herk margin recipe does not account for rotation and shape variation. As per definition the group mean error (µ) should be zero over the course of treatment and any discrepancy is disregarded from the margin calculation. The margin recipe of Stroom et al. is based on coverage probability and defined as $M_{PTV}=2.0\sum+0.7\sigma$ which is very similar to van Herk’s formula. They state that the systematic error multiplied by 2.0 SD is enough to fulfil their dose coverage requirement, but their margin recipe is limited to prostate, cervix and lung cancer cases only. Another drawback is that the penumbra width is excluded from the formula. However, they describe that rotational uncertainties are included in their safety margin which results in an anisotropic expansion. In the present study Stroom et al. and van Herk et al. margin recipes were both used to calculate the CTV-to-PTV extension, but were considered as a lower limit only. [30,37]
